# Prevalence of and Risk Factors for Adolescent Obesity in Southern Appalachia, 2012

**DOI:** 10.5888/pcd11.140348

**Published:** 2014-12-18

**Authors:** Liang Wang, Deborah L. Slawson, George Relyea, Jodi L. Southerland, Youfa Wang

**Affiliations:** Author Affiliations: Deborah L. Slawson, Jodi L. Southerland, College of Public Health, East Tennessee State University, Johnson City, Tennessee; George Relyea, School of Public Health, University of Memphis, Memphis, Tennessee; Youfa Wang, School of Public Health and Health Professions, University at Buffalo, State University of New York, Buffalo, New York.

## Abstract

The objective of this study was to examine weight status among southern Appalachian adolescents and to identify risk factors for obesity. We analyzed baseline data from the Team Up for Healthy Living study in 2012. Overall, 19.8% of the sample was overweight, and 26.6% was obese. Boys had higher rates of overweight/obesity than girls (50.5% vs 42.3%). Being male (odds ratio [OR] = 1.79; 95% confidence interval [CI], 1.39–2.29), having a mother with a high school education or less (OR = 1.39; 95% CI, 1.05–1.83), or having a father with a high school education or less (OR = 1.57; 95% CI, 1.17–2.09) was associated with a higher prevalence of obesity and a higher body mass index *z* score (β = 0.131, 0.160, and 0.043, respectively, *P* < .05). Parental education could be used to identify adolescents with a higher likelihood of obesity.

## Objective

The Southern Appalachian region has one of the highest rates of obesity in the United States (1). People living in Appalachia also have a larger burden of chronic diseases such diabetes and cancer than other areas (1–3). Research on obesity risk factors among adolescents is critical because nearly three-fourths of obese adolescents become obese adults (4). Few studies have used a large sample to examine the prevalence of and risk factors for obesity among Appalachian youths. The objective of our study was to examine the prevalence of obesity and to determine its risk factors among Southern Appalachian adolescents.

## Methods

We used baseline data from the first wave (n = 544) and second wave (n = 965) of Team Up for Healthy Living (5), a cluster-randomized trial targeting obesity prevention through a school-based, cross-peer intervention among adolescents in Southern Appalachia (mean age, 14.9 years). Students in Lifetime Wellness classes were enrolled in this trial in 2 recruitment waves (January–February and August–September 2012).

Study exclusion criteria included the following: enrollment in another weight-management program; having a diagnosed eating disorder such as anorexia nervosa or bulimia nervosa; having an underlying condition affecting weight status, such as hypothyroidism, Cushing’s syndrome, or chronic steroid use; having dietary or physical activity restrictions, such as those recommended for adolescents who have hypertension, diabetes, or severe orthopedic problems; and pregnancy.

The overall participation rate for the study was 91.2% (1,509/1,654). The study was approved by the institutional review board at East Tennessee State University. All students provided assent, and parents provided passive consent.

We determined weight status by using the 2000 Centers for Disease Control and Prevention growth charts and data based on direct measurement of height and weight: overweight was defined as a body mass index (BMI) in the 85th to 95th percentile; obesity was defined as a BMI greater than the 95th percentile (6). Data on student demographics, including age, sex, race, annual family household income, and level of education for each parent, were obtained through a standardized questionnaire.

Linear and logistic mixed models were fit for identifying risk factors, with BMI *z* score and adolescent overweight and obesity as outcomes. To control for the intercorrelation of students’ outcomes within a class or school, we included those factors as random effects. All data analyses were performed using SAS version 9.2 (SAS Institute Inc).

## Results

Most (93.4%) study participants were white (Table 1). Nearly half of mothers (48.6%) and 36.1% of fathers had some college or more. Overall, the prevalence of overweight was 19.8%, and the prevalence of obesity was 26.6%. Boys had a higher prevalence of obesity than girls (32.3% vs 20.8%) but a lower prevalence of overweight (18.2% vs 21.5%).

**Table 1:** Characteristics of Adolescent Participants, by Sex, in the Team Up for Healthy Living Project, Southern Appalachia, 2012

Characteristic	Overall^a^ (n = 1,509)	Boys (n = 765)	Girls (n = 744)
**Age, mean (SD), y**	14.9 (0.7)	14.9 (0.8)	14.8 (0.7)
**Sex, %**			
Male	49.3	—	—
Female	50.7	—	—
**Race/ethnicity, %**			
American Indian or Alaska Native	1.0	1.1	0.8
Asian	0.3	0.4	0.1
Black or African American	0.8	0.8	0.7
Hispanic or Latino	2.7	2.7	2.7
Native Hawaiian or Other Pacific Islander	0.1	0	0.1
White	93.4	92.9	93.9
Other	1.9	2.2	1.7
**Mother’s highest level of education, %**			
Less than high school	6.0	5.3	6.8
High school graduate or GED	29.1	30.1	28.1
Some college	21.5	21.2	21.9
College degree	27.1	26.8	27.4
Do not know	16.3	16.6	15.9
**Father’s highest level of education, %**			
Less than high school	8.5	8.2	8.8
High school graduate or GED	33.3	34.1	32.5
Some college	15.2	16.8	13.6
College degree	20.9	21.1	20.7
Do not know	22.1	19.8	24.4
**Annual family household income, %**			
<$20,000	3.9	4.1	3.6
$20,000–$44,999	7.6	9.7	5.5
$45,000–$74,999	7.7	8.9	6.5
≥$75,000	8.8	11.5	6.1
Do not know	72.0	65.8	78.3
**ICC^a^ (*P* value)**			
Weight	0.012 (.09)	0.005 (.35)	<0.001 (.48)
Height	0.022 (.03)	0.029 (.08)	0.012 (.26)
BMI	0.008 (.36)	0.010 (.24)	0^c^
**BMI measures, mean (SD)**			
BMI	24.5 (5.7)	24.8 (5.9)	24.2 (5.5)
Standardized BMI *z* score^d^	0.87 (1.04)	0.94 (1.12)	0.81 (0.95)
BMI percentile^d^	73.0 (26.5)	73.5 (27.7)	72.4 (25.3)
**Weight status,^e^ %**			
Underweight	1.1	1.5	0.8
Healthy weight	52.5	48.1	56.9
Overweight	19.8	18.2	21.5
Obese	26.6	32.3	20.8

Abbreviations:—, not available; BMI, body mass index; GED, General Education Development; ICC, intraclass correlation coefficient; SD, standard deviation.

a Data were missing in overall sample for the following: grade (n = 46), race/ethnicity (n = 48), mother’s education (n = 79), father’s education (n = 85), annual family household income (n = 54), and measured weight (n = 18).

b ICC for classes nested within schools; ICC defined as σ^2^B/(σ^2^W + σ^2^B), where σ^2^B is the between-subjects (classes) variance and σW^2^ is the within-subjects variance. Variance components were estimated by a mixed linear model in SAS version 9.2 (SAS Institute Inc).

c Not estimable; BMIs varied randomly between students’ classes.

d Calculated according to Centers for Disease Control and Prevention 2000 growth charts (6).

e Measured weight status categories were assigned via age- and sex-specific BMI percentile scores based on the Centers for Disease Control and Prevention 2000 growth charts (6).

Being male, having a mother with a high school education or less, or having a father with a high school education or less was associated with a higher likelihood of obesity (being male, OR = 1.79 [95% CI, 1.39–2.29]; mother’s education, OR = 1.39 [95% CI, 1.05–1.83]; father’s education (OR = 1.57 [95% CI, 1.17–2.09]) and a higher BMI *z* score (being male, β = 0.131 [*P* = .02]; mother’s education, β = 0.160 [*P* = .008]; father’s education, β = 0.043 [*P* = .003]) (Table 2). When stratified by sex, only a low level of paternal education predicted BMI *z* scores for boys, whereas only a low level of maternal education predicted BMI z scores for girls. A low level of maternal education (OR = 1.51; 95% CI, 1.03–2.21) and a low level of paternal education (OR = 1.70; 95% CI, 1.16–2.50) were associated with a higher likelihood of obesity for boys, but not girls.

**Table 2:** Adjusted Odds Ratios (ORs) and 95% Confidence Intervals (CIs) for Adolescent Overweight and Obesity and Regression Coefficient for BMI *z* Score, Southern Appalachia, by Sex, 2012

Characteristic	Overall			Boys			Girls		
	Overweight^a^, OR (95%CI)	Obesity^b^, OR (95% CI)	BMI z Score^c^, β	Overweight^a^, OR (95% CI)	Obesity^b^, OR (95% CI)	BMI z Score^c^, β	Overweight^a^, OR (95% CI)	Obesity^b^, OR (95% CI)	BMI z Score^c^, β
**Age**	0.99 (0.98–1.01)	1.01 (1.00–1.03)	0.004	0.99 (0.85–1.15)	1.01 (0.99–1.03)	0.006	1.00 (0.97–1.02)	1.01 (.99–1.03)	−0.001
**Sex**									
Girls	1.0 [Referent]			—			—		
Boys	0.98 (0.75–1.29)	1.79 (1.39–2.29)	0.131^d^	—			—		
**Race**									
Nonwhite	1.0 [Referent]			1.0 [Referent]			1.0 [Referent]		
White	1.37 (0.83–2.24)	0.96 (0.64–1.44)	−0.002	2.16 (0.01–4.97)	1.01 (0.59–1.73)	0.133	1.01 (0.54–1.89)	0.89 (0.48–1.65)	−0.142
**Annual family household income**									
≥$45,000	1.0 [Referent]			1.0 [Referent]			1.0 [Referent]		
Unknown	1.05 (0.72–1.52)	0.94 (0.67–1.32)	−0.040	1.28 (0.76–2.15)	1.17 (0.76–1.80)	0.030	0.81 (0.47–1.41)	0.83 (0.47–1.49)	−0.095
<$45,000	0.83 (0.47–1.45)	1.36 (0.86–2.15)	0.029	1.03 (0.49–2.16)	1.38 (0.78–2.46)	0.009	0.62 (0.26–1.47)	1.35 (0.62–2.91)	0.055
**Mother’s education**									
Some college or more	1.0 [Referent]			1.0 [Referent]			1.0 [Referent]		
Unknown	0.81 (0.56–1.17)	0.94 (0.67–1.31)	−0.006	0.87 (0.52–1.46)	1.12 (0.72–1.73)	−0.018	0.75 (0.45–1.26)	0.72 (0.43–1.23)	0.001
High school or less	1.12 (0.82–1.52)	1.39 (1.05–1.83)	0.160^e^	0.99 (0.63–1.55)	1.51 (1.03–2.21)	0.158	1.24 (0.82–1.88)	1.29 (0.85–1.96)	0.171^f^
**Father’s education**									
Some college or more	1.0 [Referent]			1.0 [Referent]			1.0 [Referent]		
Unknown	0.99 (0.70–1.40)	1.04 (0.75–1.45)	0.786	1.13 (0.68–1.87)	1.16 (0.75–1.82)	0.065	0.88 (0.55–1.42)	1.04 (0.63–1.71)	0.032
High school or less	1.12 (0.82–1.54)	1.57 (1.17–2.09)	0.043^g^	1.10 (0.70–1.74)	1.70 (1.16–2.50)	0.265^h^	1.14 (0.74–1.75)	1.51 (0.96–2.38)	0.117

Abbreviations: BMI, body mass index; — , not available.

a Binary mixed model logistic regression was used, with overweight (excluding obesity) relative to normal weight as an outcome variable.

b Binary mixed model logistic regression was used, with obesity relative to normal/underweight as an outcome variable.

c Linear mixed model was used, with BMI *z* score as an outcome variable. Estimates evaluated at mean levels.

d *P* = .02.

e *P* = .008.

f *P* = .03.

g *P* = .003.

h *P* = .005.

We found similar results for the overall sample when we combined data for overweight and obesity. When stratified by sex, only lower levels of maternal and paternal education were associated with overweight and obesity among boys.

## Discussion

Consistent with a previous study (7), we observed a high prevalence of overweight and obesity among Appalachian adolescents, and boys were at higher risk for obesity than girls. We also found that parental education, but probably not family income, was associated with obesity. Parental education may be a more important factor than family income and should be considered in developing future interventions in Appalachia. The relationship between socioeconomic status (SES) and obesity is complex, varying by population, and may change over time (8). A recent study suggests that although obesity rates are declining among adolescents in households with higher SES, they continue to increase among young people in households with lower SES (9).

Among adolescent boys, lower levels of education among mothers and fathers predicted a higher likelihood of obesity, whereas among girls, only a lower level of education among mothers was predictive. In contrast to the findings of our study, other studies have reported that the father’s level of education was a better predictor of obesity among adolescents than the mother’s education (10). Our findings further support the concept that household education level is important for understanding health disparities.

Appalachia has a population of approximately 25 million, 42% of whom live in rural areas; only 20% of the national population lives in rural areas (11). Parents in rural areas may face unique challenges in maintaining a healthy living environment for their children.

This study has limitations. The large percentage of “do not know” responses to the question on annual family income level decreases the power of the study to detect possible differences. Second, our study sample is not representative of the entire Appalachian region. Despite these limitations, our study adds important findings to the literature on adolescent obesity in an understudied population. It suggests that parental education could be used to help identify adolescents at high risk for obesity in the target population. Future research using longitudinal data are warranted.
